# Percutaneous treatment of abdominal aortic aneurysm and aortic valve stenosis with ‘staged’ EVAR and TAVR: a case series

**DOI:** 10.1186/s13019-023-02338-7

**Published:** 2023-07-13

**Authors:** Massimo Medda, Francesco Casilli, Marta Bande, Mattia Glauber, Maurizio Tespili, Silvia Cirri, Francesco Donatelli

**Affiliations:** 1 Clinical and Interventional Cardiology Unit, Cardio-Thoracic Center, IRCCS Ospedale Galeazzi-Sant’Ambrogio, Gruppo San Donato, Via Cristina Belgioioso, 173, Milan, Italy; 2 Istituto Clinico Sant’Ambrogio, Milan, Italy; 3 IRCCS Ospedale Galeazzi-Sant’Ambrogio, University of Milan, Milan, Italy; 4 Department of Minimally Invasive Cardiac Surgery, IRCCS Ospedale Galeazzi-Sant’Ambrogio, Gruppo San Donato, Milan, Italy; 5 Department of Anesthesia and Intensive Care, IRCCS Ospedale Galeazzi-Sant’Ambrogio, Gruppo San Donato, Milan, Italy; 6 Medical Affairs EMEA, Boston Scientific Corporation, Milan, Italy

**Keywords:** Abdominal aortic aneurysm, EVAR, Aortic valve stenosis, Transcatheter aortic valve replacement, TAVR

## Abstract

Symptomatic aortic valve stenosis (AS) and abdominal aortic aneurysm (AAA) are critical clinical conditions, increasingly more prevalent with aging of the population. Calcific aortic stenosis is the most common structural cardiac disease in the elderly population, and medical management of severe aortic stenosis of the elderly population is associated with poor outcomes as compared to surgical treatment. Transcatheter aortic valve replacement (TAVR) is a treatment of choice in inoperable, often elderly, patients with symptomatic severe AS and in intermediate-to-high surgical risk patients. It is not yet clarified the incidence of AAA and its impact on procedural and clinical outcomes among patients undergoing TAVR. It is known that after AS resolution with aortic valve replacement or TAVR there is an increase in blood pressure that increases the risk of dissection or abdominal aortic aneurysm rupture if AAA repair is delayed. The purpose of this report is to describe the anatomical details and technical and procedural considerations when proposing totally endovascular strategies dedicated to the treatment of patients with AS and AAA.

**Supplementary Information:**

The online version contains supplementary material available at 10.1186/s13019-023-02338-7.

## Introduction

Symptomatic aortic valve stenosis (AS) in intermediate-to-high surgical risk patients is currently treated with transcatheter aortic valve replacement (TAVR) (Class I indication according to international guidelines) [1, 2]. The prevalence of AS is approximately 4.6% in patients aged over 75 years [3], while abdominal aortic aneurysm (AAA) has a prevalence of 5.9% in men over 80 years of age [4]. Cases of concomitant severe AS and AAA requiring repair are not rare. Furthermore, the demographics of the population are changing and it is expected that octogenarians and nonagenarian patients will quadruple by year 2050 [5]. The first randomized TAVR trials excluded patients with significant vascular diseases and aortic aneurysms [1, 6]. The presence of an AAA could impact the outcome of TAVR increasing any potential vascular complications (such as aortic aneurysm rupture, aortic dissection, peripheral embolization of thrombotic material) as improved systolic pressure could increase the risk of aneurysm rupture. We present our ‘tailored’ interventional approach, discussed and approved by the local Heart Team for each patient, that planned the endovascular correction of AAA as the first step followed by ‘staged’ TAVR.

## Methods

Starting from July 2015 and up to July 2021, all consecutive patients with symptomatic AS treated with a TAVR procedure at the Cardiothoracic Centre, Istituto Clinico Sant'Ambrogio, Milan, Italy were included in the Institutional TAVR database. We analyzed the demographic, clinical, procedural and in-hospital and 30 days outcomes of the patients suffering from significant abdominal aortic aneurysm (AAA) with maximum diameter ≥ 40 mm. A team of cardiologists, interventional cardiologists, cardiac surgeons, and anesthesiologist (‘Heart Team’) participated in the procedural planning for all patients. Consensus to proceed with endovascular aortic aneurysm repair (EVAR) followed by ‘staged’ TAVR was reached after Heart Team discussion. Informed consent was obtained from all patients before the procedure. Transfemoral EVAR and TAVI were performed under local anesthesia and mild sedation or general anesthesia according to patient's tolerance and procedural complexity. Length of stay was calculated from EVAR or TAVR procedure (day 0) to day of discharge. All procedures were performed by a ‘single’ interventional cardiologist team (M.M., F.C., M.B.) and the treating interventional cardiologist was contacted for long-term follow-up information. All events, including device success, were classified according to the Valve Academic Research Consortium-2 (VARC-2) criteria [7]. Clinical follow-up included all-cause mortality, cardiovascular mortality, disabling and non-disabling stroke (according to the modified Rankin scale) and cardiovascular (CV)-related hospitalization (heart failure, transient ischemic attack [TIA], arrhythmia, myocardial infarction). Transthoracic echocardiography was performed before and after TAVR. Postprocedural transthoracic echocardiography was performed the same day of procedure and repeated at discharge. Post-procedural paravalvular leak (PVL) were assessed by experienced echocardiographers according the Valve Academic Research Consortium-2 (VARC-2) criteria and classified as absent, mild, moderate and severe [7]. Pre-procedural Multislice Computed Tomography (MSCT) was performed using a 64-slice scanner (SOMATOM Definition AS, Siemens Healthcare s.r.l., Germany). We proceeded to reconstruct the images using either OsiriX DICOM Viewer (Pixmeo SARL, Switzerland) or 3mensio Structural Heart software (Pie Medical Imaging, Netherlands), according to multiple time windows between 0 and 100% of the R–R period. The evaluation of the aortic root and of the annulus was performed in the systolic phases. Categorical variables are expressed as numbers with percentages, while continuous variables are presented as mean ± standard deviation.

## Case series

During the study period we treated 533 patients with symptomatic AS with a TAVR procedure. We describe the experience of n = 5 consecutive patients presenting association of AS and AAA, all males, treated exclusively with percutaneous procedures (EVAR and subsequently TAVR). Baseline characteristic are reported in Table 1. The mean age (± SD) was 76.6 years (± 8.01 years), the mean STS score and Euroscore II were respectively 3058 ± 0.9 and 4.370 ± 0.7. All patients were in NYHA class II at admission. Baseline pre-TAVR echocardiographic and MDCT findings were summarized in Tables 2 and 4. We proposed a delay time between EVAR and TAVR at least 30 days, in order to prevent kidney damage. The average time actually elapsed between the two procedures was 4.8 months. The reported time interval reflects more the waiting list for TAVR intervention in our Cardio-Thoracic Center and the patient's programming preferences, rather than a clinical choice. GORE Excluder prosthesis (W. L. Gore & Associates, Flagstaff, Arizona) were used in n = 4 EVAR procedures, in one patient in association with a GORE EXCLUDER Iliac Branch Endoprosthesis (IBE) to treat an significant iliac bifurcation involvement, and n = 1 a GORE TAG Conformable Thoracic Stent Graft was implanted. Two ‘self-expanding’ valves (n = 1 Portico, Abbott and n = 1 Evolut R, Medtronic) and three ‘balloon-expanding’ valves (n = 2 Myval, Meril and n = 1 Sapien 3, Edwards) were used for the TAVR procedures. Transfemoral access was used in 100% of patients and a single complex EVAR procedure (Case 1) was performed under general anesthesia (Table 2). Unlike the commonly applied technique in our center, preferring TAVR with sheathless approach whenever possible, in patients with previous EVAR we used in all cases an introducer through the less tortuous iliac and femoral arteries under fluoroscopic guidance to avoid stent deformation or displacement of the EVAR prosthesis using a brachiofemoral through-and-through wire technique in one case [8]. Pre-dilatation and post-dilatation were performed respectively in 3 and 2 patients (Table 4). The main vascular access protection with a crossover 0.018″ guidewire placement via contralateral femoral access was utilized in one case. We used in n = 3 cases a protection through femoro-femoral ipsilateral access and n = 1 case through omeral artery. All procedures were successful and patients were discharged 6.6 days (± 4.72) after the EVAR procedure and 7 days (± 3.53) after the TAVR procedure. Procedural time and hospital length of stay were comparable to those of either procedure done separately. A major vascular complication occurred in one patient and was corrected percutaneously by implantation of a covered stent (Case 2). A permanent pace-maker (PPM) implantation was required after the late occurrence of a third-degree atrioventricular block (AV block) (Case 4) (Table 5). No cases of periprocedural stroke, coronary occlusion or myocardial infarction occurred. There was no 30-day mortality (Table 5).

**Table 1:** Patients baseline characteristics

Case	Age (years)	Gender	BSA, m^2^	BMI (kg/m^2^)	NYHA class	GFR (mL/min)	LVEF (%)	Comorbidity	CAD	STS-PROM (%)	Logistic Euroscore II (%)
1	77	M	1.8	30.08	II	41	55	AH, CKD, ex-smoker, dyslipidemia, Thrombocytopenia (mild) Chronic multifactorial anemia Right carotid stenosis 80%; left carotid stenosis 60%	Prior NSTEMI Critical CX stenosis treated with PCI and DES implantation CTO of RCA	1.999	4.21
2	75	M	2.0	24.69	II	26	55	AH, dyslipidemia, ex-smoker CKD, PAD Paroxysmal AF	Prior LAD stenting (2007) CTO of RCA	3.62	3.36
3	64	M	2.1	30.67	II	85	36	Ex-smoker Dyslipidemia Obesity Severe BPCO (Gold 4)	Prior antero-septal STEMI Multiple coronary stenting Prior CABG (× 4) and LV aneurysm correction (LV plication)	2.449	4.91
4	84	M	1.8	28.58	II	70	60	AH, dyslipidemia Asymptomatic right carotid stenosis (60–65%)	Significant CX and LM stenosis treated with DES implantation	4.283	4.05
5	83	M	1.87	27.28	II	72	35	AH, ex smoker, dyslipidemia COPD Permanent AF (OAC) Bilateral carotid artery stenosis (50–55%)	Significant stenosis of distal RCA	2.937	5.32

*AH* arterial hypertension, *AF* atrial fibrillation, *AVA* aortic valve area, *BSA* body surface area, *BMI* body mass index, *CABG* coronary artery bypass grafting, *CAD* coronary artery disease, *COPD* chronic obstructive pulmonary disease, *CTO* chronic total occlusion, *CKD* chronic kidney disease (eGFR < 60 mL/min/1.73 m^2^), *CVA* cerebrovascular accident, *CX* circumflex coronary artery, *DES* drug eluting stent, *DM* diabetes mellitus, *LAD* left anterior descending artery, *LFLG* low flow low gradient, *LM* left main, *LV* left ventricle, *LVEF* left ventricle ejection fraction, *NYHA* New York Heart Association, *OAC* oral anticoagulant, *PAD* peripheral arterial disease, *PCI* percutaneous coronary intervention, *RCA* right coronary artery, *STEMI* ST elevation myocardial infarction, *STS-PROM* Society of Thoracic Surgeons-Predicted Risk of Mortality

**Table 2:** EVAR planning and procedure

Case	Aortic diameter: proximal implantation site (mm)	Aortic diameter: 15 mm inferior to proximal implantation site (mm)	Maximum outer aneurysm diameter (mm)	Right common iliac diameter (mm)	Left common iliac diameter (mm)	Right external iliac diameter (mm)	Left external iliac diameter (mm)	Right common femoral diameter (mm)	Left common femoral diameter (mm)	Aortic neck length (mm)	Anesthesia	Contrast (mL)	Fluoroscopy time (min)	PCI during EVAR
1	28.1	27.0	77 × 71	24 × 27	19.5	10	10	12	14	40	General	170	85.25	No
2	21.3	20.9	40	10.5 × 12.4	17.5 × 10	8 × 6.8	6.5 × 6.9	10.7	10.5	20	Local	140	28.4	DES LAD
3	19.7	20.2	70 × 70	16.5 × 19	11 × 11	7 × 6	7 × 6.7	7	9.5	30	Local	100	24.11	No
4	22	25.3	40	13.4 × 12.5	15 × 13.5	9.5 × 8.8	9.6 × 8.7	9.2	9	15	Local	230	39.51	No
5	21.1	20	56 × 52	11 × 13.2	11.2 × 16	7.9 × 7.2	8 × 8.2	8.5	8	40	Local	125	19.04	No

All CT data are derived with post-processing analysis with dedicated software (OsiriX or 3mensio)

*DES* drug eluting stent, *LAD* left descending artery

### Case 1 (Tables 1, 2, 3, 4 and 5, Fig. 1, Case 1 panels A–D, Additional file 1: Movie S1, Additional file 2: Movie S2, Additional file 3: Movie S3, Additional file 4: Movie S4 and Additional file 5: Movie S5)

**Fig. 1:**
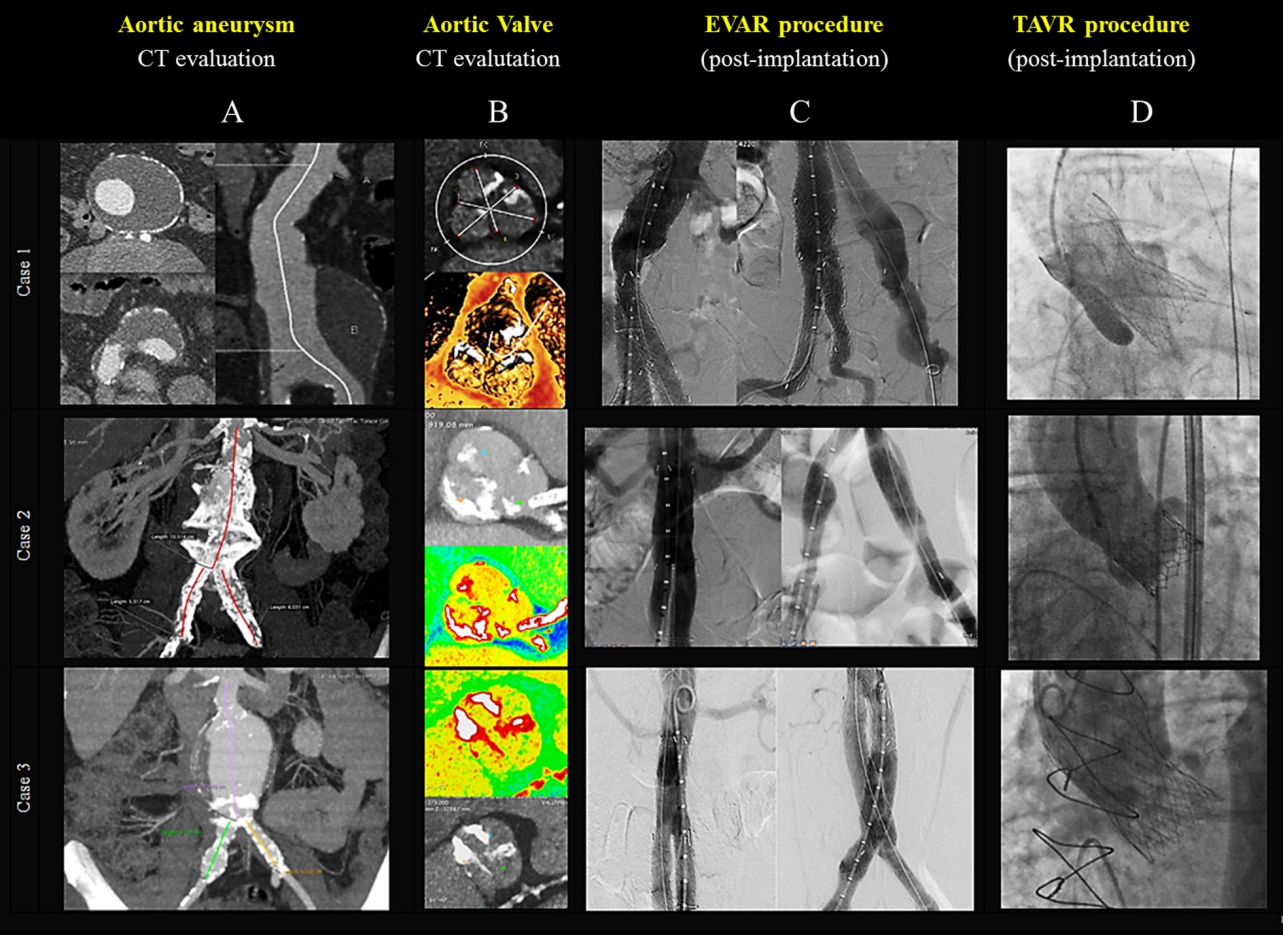
Cases 1–3 pre-procedural images of computed tomography (CT) angiography with tridimensional reconstruction of abdominal aortic aneurysm (panel A) and anatomy of the aortic valve (panel B). Post-procedural EVAR (panel C) and TAVR (panel D) results

A 77-year-old male, symptomatic for exertional dyspnea (NYHA Class II) and effort angina, was evaluated for severe AS. Transthoracic echocardiography (TTE) showed a calcified tricuspid aortic valve with valve area of 0.9 cm^2^, peak and mean gradients of 74 mmHg and 43 mmHg, respectively, and mild left ventricular hypertrophy with normal ejection fraction. The baseline characteristics and comorbidities are presented in Table 1. The CTA of the abdominal aorta showed a large fusiform aneurysm, with the largest diameter of 77 mm, mural thrombus, and proximal neck of 28.1 mm with significant involvement of iliac arteries (Table 2) (Fig. 1, Case 1, panel A). The computed tomography angiography (CTA) assessment of the tricuspid aortic valve apparatus is shown in Fig. 1, Case 1, panel B. The patient presented a STS PROM of 1.999% (Society of Thoracic Surgeons—STS—PROM predicted risk of mortality) and 4.21% according to EuroSCORE II. The EVAR procedure was performed under general anesthesia with bilateral percutaneous femoral approach (16F introducer on the right and 18F introducer on the left; ‘preclosing’ with Perclose Proglide × 2). Treatment of the iliac aneurysm was performed using a dedicated GORE prosthesis (GORE Excluder IBE—“Iliac Branch Endoprosthesis”, W. L. Gore & Associates, Flagstaff, Arizona) with reconstruction of the iliac bifurcation (CEB 23-12-10, hypogastric stent 16-12-07). We performed the EVAR placing a bifurcated GORE Excluder endoprosthesis (RLT 31-14-13, PLC 23-12-00) and a prosthetic connection between the two bifurcated prosthesis (bridge PLC 27-12-00), finally optimizing the sealing dilating the proximal and distal segments and the overlaps with an elastomeric balloon. The final angiography revealed a correct positioning and expansion of the prosthetic elements, the exclusion of both aortic and iliac aneurysms and the absence of ‘endoleak’ (Fig. 1, Case 1, panel C). Femoral bilateral hemostasis with suture-based closure device (Proglide × 2) was obtained. The overall procedural fluoroscopic time was 85.25 min. The total amount of contrast administrated was 170 mL (Tables 2 and 3). A significant underexpansion of metallic frame of the hypogastric side branch was detected by a computed tomography (without administration of iodinated contrast medium) performed before the discharge. A ‘kissing balloon’ dilatation of the iliac bifurcation between the internal and external iliac arteries was therefore performed with a double arterial approach (left omeral artery and right femoral artery). Two months later, the TAVR procedure was performed with a 27 mm size PORTICO aortic valve direct implantation (Portico valve, Abbott Vascular) (Table 4). The 19F St. Jude Introducer was advanced via a brachiofemoral through-and-through wire technique (from the right radial artery through the aortic endoprosthesis and to the femoral artery). The TAVR procedure was uneventful. Hemostasis of the main vascular access was obtained with double Proglide devices (preclosing method) and ‘endoclamp’ with an 8 mm peripheral balloon dilatation. The vascular protection with a 0.018″ guidewire was achieved through the left omeral artery (7F introducer). The final angiography showed a mild paravalvular leakage. The pre-discharge TTE documented a transvalvular mean gradient of 9 mmHg with preserved LV function. The procedural fluoroscopic time was 29,47 min. The total amount of contrast administrated was 180 mL (Tables 4 and 5) (Fig. 1, Case 1, panel D).

**Table 3:** EVAR prosthesis and procedure

Case	Gore Excluder	Gore TAG endograft	Trunk ipsilateral leg endoprosthesis	Contralateral leg endoprosthesis	Aortic extender endoprosthesis implantation	IBE (Iliac branch Endoprosthesis)	Aneurysmatic sac embolization	Polar arteries exclusion	Right Introducer dimension (Fr)	Right Vascular Access Closure	Left Introducer dimension (Fr)	Left Vascular Access Closure
1	Yes	No	RLT 31-14-13	PLC 23-12-00	No	Yes (planned) CEB 23-12-10 Hypogastric stent 16-12-07 Bridge PLC 27-12-00	No	No	16	Proglidex2 preclosing	18	Proglidex2 preclosing
2	Yes	No	RLT 26-14-16	PLC 12-14-00	No	No	No	Yes (planned)	16	Proglidex2 preclosing	12	Proglidex2 preclosing
3	Yes	No	RLT 23-14-16	PLC 20-14-00	No	No	No	No	12	Proglidex2 preclosing	16	Proglidex2 preclosing
4	Yes	No	RLT 28-14-14	PLC 16-12-00	Yes (unplanned)	No	No	No	18	Proglidex2 preclosing	12	Proglidex1 preclosing
5	No	Yes	TGM 26-21-20	No	No	No	Yes (n = 2 coils 15 × 250)	Yes (planned)	20	Proglidex2 preclosing	12	Proglidex1 preclosing

**Table 4:** TAVR planning and procedure

Case	Valve anatomy	TTE AVA (cm^2^)	TTE transvalvular gradient peak/mean (mmHg)	CT aortic annulus area (mm^2^)	CT aortic annulus perimeter (mm)	CT perimeter derived diameter (mm)	CT LVOT area (mm^2^)	CT LVOT perimeter (mm)	THV	Contrast (mL)	Fluoroscopy time (min)	Introducer main vascular access	through-and-through wire technique (radial-femoral)	Access protection	Local anesthesia	Main access closure	Pre-dilatation	Post-dilatation
1	Tricuspid	0.9	74/43	444.9	76.25		276.8	65.8	PORTICO n.27	180	29.47	19F St. Jude	Yes	Left omeral artery	Yes	Proglidex2 preclosing	No	No
2	Tricuspid	0.8	75/45	408.3	73.3	23.3	268.1	62.1	SAPIEN 3 n. 23	90	20.35	14F eSheath#	No	Omolateral	Yes	Prostar XL preclosing	No	Yes (23 mm)
3	Bicuspid type 1 L-R with calcific raphe	N/A	84/50	492.3	82.5	26.7	457.1	79	EVOLUT R 34 mm	250	37.26	20F Gore Dryseal	No	Omolateral	Yes	Proglidex2 preclosing	Yes (23 mm)	Yes (24 mm)
4	Tricuspid	0.9	70/40	428.8	74.6	23.7	430.2	74.7	MYVAL 24.5 mm	120	18.14	Python 14F#	No	Omolateral	Yes	Prostar XL preclosing	Yes (20 mm)	No
5	Tricuspid	0.8	37/22*	462.7	76.7	24.4	433	74.7	MYVAL 26 mm	150	20.41	Python 14F#	No	Crossover (femoral contralateral)	Yes	Proglidex2 preclosing	Yes (20 mm)	No

*CT* computed tomography, *N/A* not available, *TTE* transthoracic echocardiogram, *THV* transcatheter heart valve, *TTWT* through-and-through

wire technique

^*^Severe LV impairment with LFLG low flow low gradient aortic valve stenosis

^#^Expandable sheath (introducers with dynamic expansion mechanism and transient sheath expansion during THV delivery)

**Table 5:** In-hospital and 30 days outcomes after TAVR procedure

Case	Device success	Post-procedural TTE transvalvular gradient peak/mean (mmHg)	PVL	LVEF (%)	Major Vascular complication VARC-2y	Femoral hematoma pseudoaneurysm	Permanent pacemaker implantation	Stroke/TIA	Periprocedural MI	Unplanned intra-procedural PCI	Stage 2 or 3 Acute kidney injury	Bleeding (BARC)	Hospitalization lenght (days)*	30 days mortaliy
1	Yes	15/9	Mild	55	No	No	No	No	No	No	No	No	13	No
2	Yes	14/7	No	55	Yes	Yes	No	No	No	No	No	No	5	No
3	Yes	16/7	Mild	45	No	No	No	No	No	No	No	No	7	No
4	Yes	20/11	No	60	No	No	Yes	No	No	No	No	No	6	No
5	Yes	18/10	No	39	No	No	No	No	No	No	No	No	4	No

*BARC* bleeding academy research consortium, *LVEF* left ventricle ejection fraction, *PVL* paravalvular leak, *TIA* transient ischemic attack, *VARC* vascular academy research consortium

^*^Length of stay was calculated from TAVR procedure (day 0) to day of discharge

### Case 2 (Tables 1, 2, 3, 4 and 5, Fig. 1, Case 2 panels A–D, Fig. 2, Additional file 6: Movie S6, Additional file 7: Movie S7, Additional file 8: Movie S8 and Additional file 9: Movie S9)

**Fig. 2:**
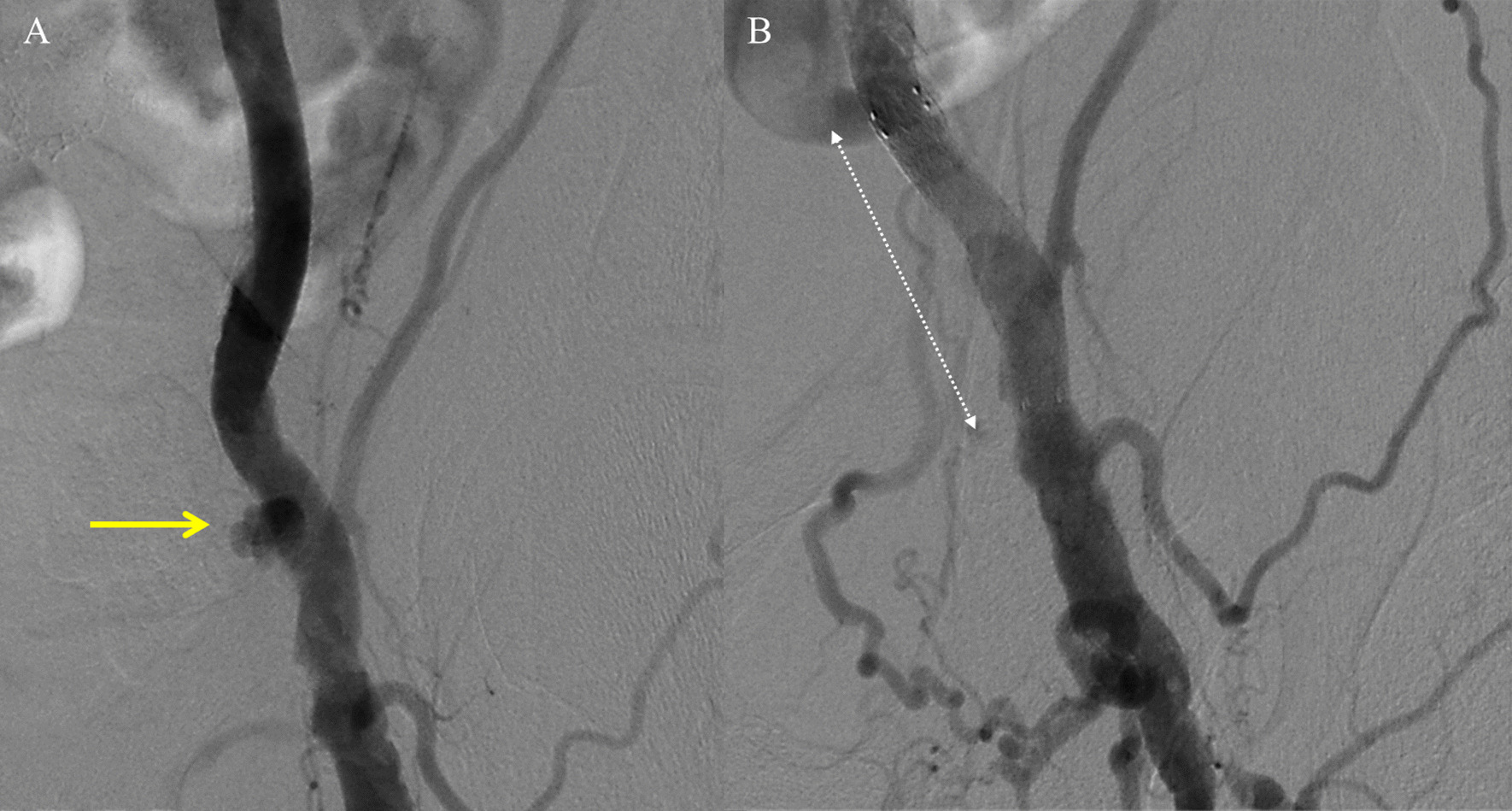
The angiography study showing the presence of a left common femoral artery pseudoaneurysm (yellow arrow, panel A) successfully treated by endovascular exclusion with GORE Viabhan 8 × 50 mm Endovascular graft implantation (dashed white line, panel B)

We present the case of an 75-year-old man, with a history of smoking, AH, dyslipidemia, paroxysmal atrial fibrillation (AF), severe CKD (GFR of 26 mL/min/1.73), coronary artery disease (CAD) treated with percutaneous coronary intervention (PCI) of the left descending artery (LAD) 10 years ago e severe peripheral artery disease (PAD) with previous right carotid artery, bilateral superficial femoral arteries and right renal artery stenting (Table 1). TTE showed a calcified tricuspid aortic valve with severe stenosis (valve area of 0.8 cm^2^, peak and mean gradients of 75 mmHg and 45 mmHg, respectively), mild left ventricular hypertrophy with normal ejection fraction (LVEF 55%) (Table 1). A pre-procedural coronary CTA examination showed significant in-stent restenosis of left descending artery and an AAA (with huge thrombotic ulceration) with maximum diameters of 40 mm and proximal neck of 20.9 mm extending to left iliac artery in the presence of multiple significant stenosis of right external iliac artery and chronic occlusion of the right internal artery (Table 2) (Fig. 1, Case 2, panel A). For conventional AVR, the predicted risk of mortality STS PROM was 3.62% and logistic EuroSCORE II 3.36%. A combined procedure of PCI and EVAR was planned. Under local anesthesia a bilateral percutaneous femoral access were obtained (16F introducer on the right and 12F introducer on the left; ‘preclosing’ with Perclose Proglide × 2) and we perform a direct implantation of DES to treat a significant in-stent restenosis of LAD (Synergy 3.0–16 mm). The EVAR was performed with the placement of bifurcated GORE Excluder endoprosthesis (RLT 26-14-16, PLC 12-14-00) with a planned small right inferior polar artery exclusion (Table 3) (Fig. 1, Case 2, panel C). The Contralateral Leg Endoprosthesis (PLC 12-14-00) extends up to the left external iliac artery achieving a ‘paving’ of the left vascular access. The final angiographic control showed the good apposition of the prosthesis and the excellent proximal and distal sealing. The overall procedural fluoroscopic time was 28.4 min. The total amount of contrast administrated was 140 mL (Table 3). Four months later, the TAVR procedure was performed with a 23 mm size SAPIEN 3 aortic valve bioprostheses implantation (SAPIEN 3 valve, Edwards Lifesciences Corporation, Irvine, CA, USA) without predilation, under rapid pacing, with planned post-dilatation with the same balloon in a more ventricular position to prevent an excessive distension of a circumferentially calcified sinotubular junction (Table 4). The TAVR was uneventful. Hemostasis of main left femoral vascular access (14F expandable eSheath, Edwards Lifesciences Corporation, Irvine, CA, USA) was obtained with a Prostar XL device (‘preclosing method’). Vascular protection with a 0.018″ guidewire was achieved through a femoro-femoral ipsilateral access. The procedural fluoroscopic time was 20,35 min. The total amount of contrast administrated was 90 mL. The pre-discharge TTE documented a transvalvular mean gradient of 7 mmHg with preserved LV function (Tables 4 and 5) (Fig. 1, Case 2, panel D). The patient presented a sudden onset of bruising and pain in the left groin region about 15 days after discharge. A ‘late’ femoral pseudoaneurysm was documented by echo-color-Doppler evaluation. The patient underwent angiography which confirmed the presence of a left common femoral artery pseudoaneurysm successfully treated by endovascular exclusion with GORE Viabahn 8 × 50 mm Endovascular graft (W. L. Gore & Associates, Flagstaff, Arizona) implantation from the right omeral arterial access (Fig. 2).

### Case 3 (Tables 1, 2, 3, 4 and 5, Fig. 1, case 3 panels A–D, Fig. 3)

**Fig. 3:**
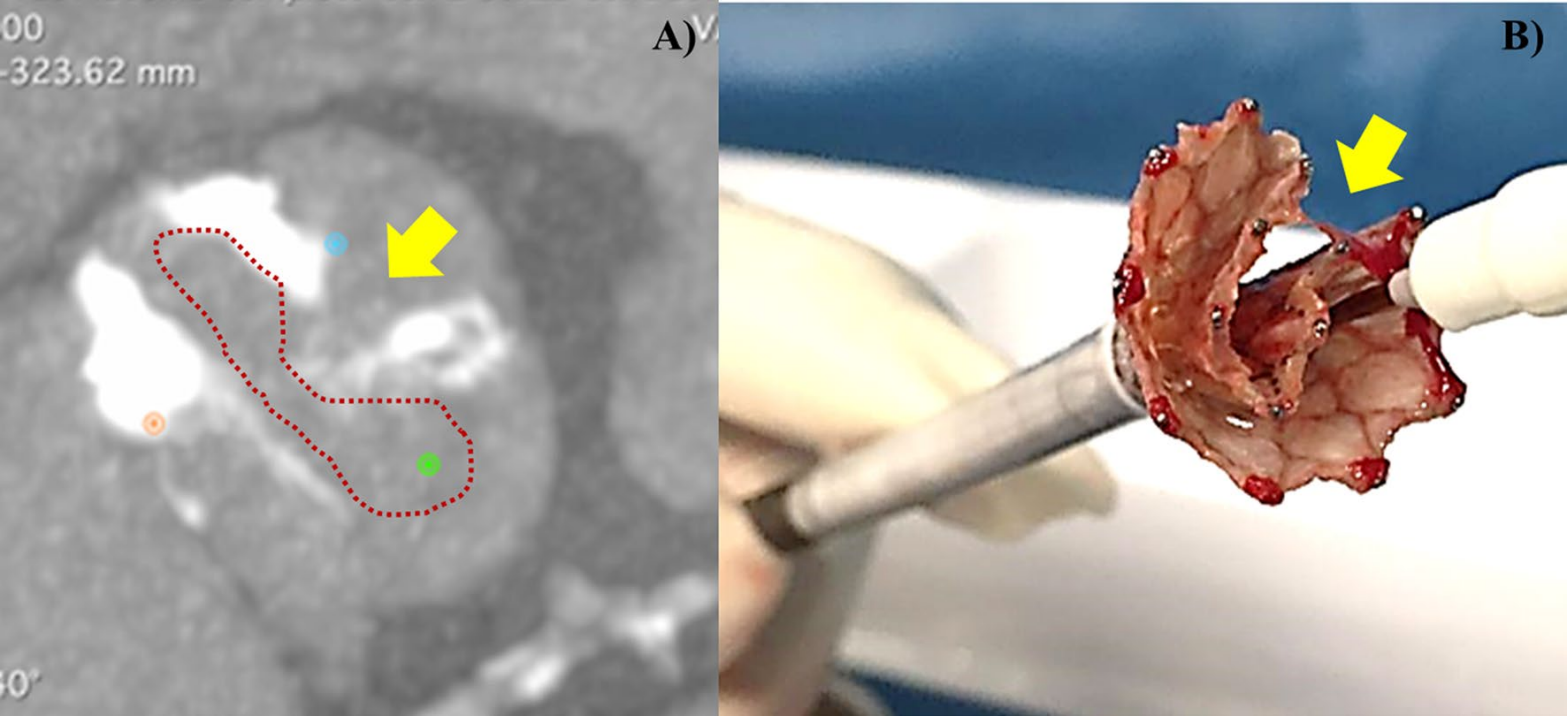
Panel A Pre-procedural images of computed tomography (CT) angiography with tridimensional reconstruction of aortic valve showed a bicuspid aortic valve (Type 1, L–R by Sievert Classification) with partially calcific raphe. The red dotted line reproduces the profile of the aortic valve orifice with evident protrusion of raphe (yellow arrow). Panel B Intraprocedural photographic image of infolded Evolut R THV with large invagination of the frame (yellow arrow) that exactly reproduces the aortic valve orifice profile as evidenced by CT (see Panel A)

This case has been previously published by our group [9]. We present the case of a 64-year-old patient, BMI 30, former smoker, suffering from severe chronic obstructive pulmonary disease (Gold 4 classification), post-myocardial infarction cardiomyopathy who underwent percutaneous and surgical revascularization (triple CABG [coronary artery by-pass graft] by left internal mammary artery on the left anterior descending artery and venous jumpgraft on the intermediate branch, obtuse marginal and right coronary artery) associated with surgical correction (‘plication’) of the apico-septal left ventricle (LV) aneurysm. The patient became symptomatic for dyspnea on exertion (New York Heart Association functional class II–III), he was evaluated by our Cardio-Thoracic Center for severe aortic valve stenosis and significant depression of the LV systolic function (LV ejection fraction 30–35%) (“low flow low gradient” AS). The coronary angiography confirmed a severe triple coronary vessel disease with patency of the arterial and the venous grafts. The pre-operative thoraco-abdominal computed tomography (CT) scan documented an aortic annulus perimeter of 82.5 mm with a very elliptical shape (minor annulus diameter 20 mm, major annulus diameter 30.6 mm), a bicuspid valve with partially calcified raphe (Type 1 L–R morphology according to the Sievers anatomical classification) [10] and asymmetric calcifications involving mostly the non-coronary cusp (Fig. 1, Case 3, panel B). Furthermore the thoraco-abdominal CT documented a 7 × 7 cm AAA below the renal arteries (Fig. 1, Case 3, panel A). The STS PROM (SAVR) was 2449%. The multidisciplinary Heart Team decision was to proceed with a completely percutaneous treatment, first the EVAR and subsequently the elective TAVR with a self-expandable transcatheter aortic heart valve (Evolut R 34 mm, Medtronic). The AAA was excluded percutaneously with a Gore Excluder AAA bifurcated Endoprosthesis (Excluder RTL 23-14-16, PLC 20-14-00; W.L. Gore and associates, Medical Products Division, Flagstaff, Arizona, USA) (Fig. 1, Case 3, panel C). The procedural fluoroscopic time was 24.11 min. The total amount of contrast administrated was 100 mL. Two months later we performed a percutaneous transfemoral TAVR under conscious sedation, through a 20F Introducer Sheath (GORE DrySeal Flex), with two Proglide “pre-closing” (Abbott Vascular, Irvine, CA) and ipsilateral ‘wire protection’ (Steel core 0.018″ guidewire). After positioning a dedicated pre-shaped guidewire (Safari 2 Small, Boston Scientific Marlborough, Massachusetts, USA) in the LV and performing fluoroscopic control of the valve (CoreValve Evolut R transcatheter valve R 34 mm, Medtronic, Minneapolis, Minnesota, USA) we proceeded with pre-dilation using a 23 mm balloon (VACS II, 23–40 mm, Osypka, Rheinfelden, Germany). During valve implantation a partial ‘re-sheathing’ was done due to a too ‘high’ position; the second attempt resulted more acceptable in terms of depth but was complicated by a rapid severe hemodynamic deterioration. Rotational fluoroscopy analysis (right anterior oblique view to left anterior oblique view projections) identified a distortion along the metallic valve frame without a clear single ‘vertical line’, but rather an ‘irregular dark line’ was identifiable longitudinally. In the suspicion of the ‘infolding phenomenon’ the valve was retracted uneventfully and the ex-vivo visual control confirmed the severe invagination of the stent frame (infolding). Interestingly, the infolded valve with large distortion of the metallic frame exactly reproduces the native aortic valve orifice profile suggesting a pathogenic role of the calcified raphe in the ‘infolding phenomenon” (Fig. 3, Panels A, B). A second Evolut R 34 mm was successfully implanted and optimized by post-dilatation with a 24 mm balloon (VACS III, 24–40 mm, Osypka, Rheinfelden, Germany) (Fig. 1, Case 3, panel D). The procedural fluoroscopic time was 37.26 min. The total amount of contrast administrated was 250 mL.

### Case 4 (Tables 1, 2, 3, 4 and 5, Fig. 4, case 4 panels A–D, Additional file 10: Movie S10 and Additional file 11: Movie S11)

**Fig. 4:**
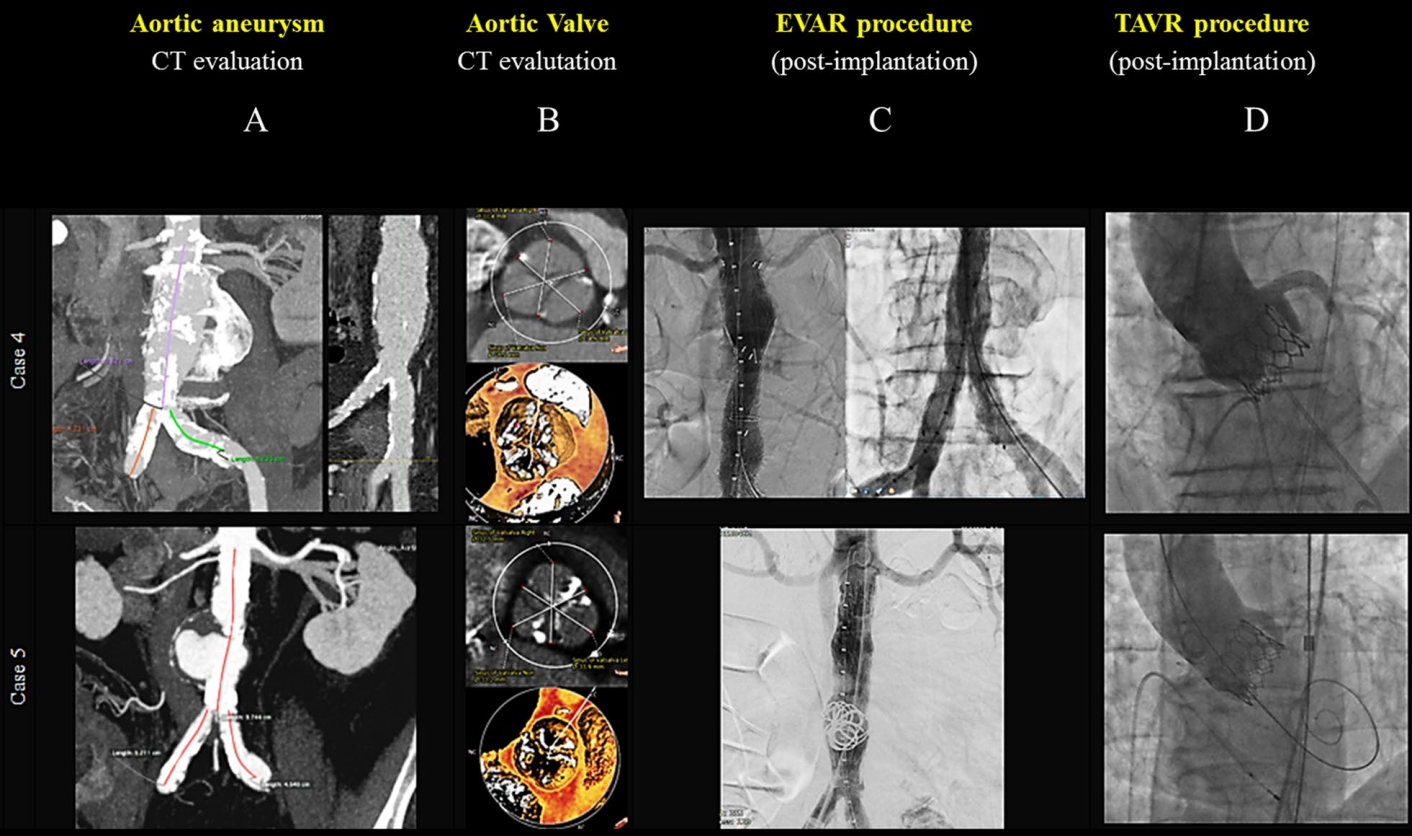
Cases 4–5 pre-procedural images of computed tomography (CT) angiography with tridimensional reconstruction of abdominal aortic aneurysm (panel A) and anatomy of the aortic valve (panel B). Post-procedural EVAR (panel C) and TAVR (panel D) results

The patient is an 84-year-old man who presented with severe symptomatic AS, AH, bilateral carotid stenosis and an saccular AAA with evidence of focal vessel dissection (Fig. 4, Case 4, panel A). The computed tomography angiography (CTA) assessment of the tricuspid aortic valve apparatus showed a severely calcified tricuspid aortic valve (Fig. 4, Case 4, panel B). For conventional AVR, his predicted STS PROM was 4.283% and logistic EuroSCORE II 4.05%. The treatment scheduled was endovascular repair of the aneurysm followed by transcatheter aortic valve replacement. The AAA was excluded percutaneously with a Gore Excluder AAA bifurcated Endoprosthesis (Excluder RTL 28-14-14, PLC 16-12-00) and a GORE Aortic cuff 28–30 was implanted to correct a type 1 Endoleak (Fig. 4, Case 4, panel C). The procedural fluoroscopic time was 39.51 min. The total amount of contrast administrated was 230 mL. Four months later, the TAVR procedure was performed with a 24.5 mm size balloon-expandable MYVAL aortic valve bioprostheses implantation (Meril Life Sciences, Vapi, India) with 20 mm balloon pre-predilation, under rapid pacing (Table 4). The introducer advancement (14F expandable Python introducer, Meril, Vapi, India) was easy and uneventfully. The correct hemostasis of main femoral vascular access was obtained with Prostar XL device (‘preclosing method’). The vascular protection with a 0.018″ guidewire was achieved through a femoro-femoral ipsilateral access. The final angiography showed a correct valve implantation with no significant residual transvalvular gradient nor paravalvular leak (Fig. 4, Case 4, panel D). The procedural fluoroscopic time was 18.14 min. The total amount of contrast administrated was 120 mL. The 3rd postoperative day the patient presented third-degree atrioventricular block (AV block) treated with dual-chamber pacemaker implantation. The pre-discharge TTE documented a transvalvular mean gradient of 11 mmHg with preserved LV function (Table 5). The patient was discharged from the hospital at the 6th postoperative day in a very satisfactory clinical condition.

### Case 5 (Tables 1, 2, 3, 4 and 5, Fig. 4, case 5 panels A–D, Additional file 12: Movie S12 and Additional file 13: Movie S13)

We present the case of a 83-year-old male patient with severe symptomatic AS, chronic obstructive pulmonary disease (COPD), CAD with involvement of the distal segments, permanent atrial fibrillation (AF) on anticoagulant therapy and bilateral carotid artery stenosis, which has been diagnosed an abdominal aortic aneurysm (AAA), 56 mm in its largest diameter, with huge ‘blister’ (Fig. 4, Case 5, panel A). TTE showed a calcified tricuspid aortic valve with low-flow low-gradient aortic valve stenosis (valve area of 0.8 cm^2^, peak and mean gradients of 37 mmHg and 22 mmHg, respectively) and reduced ejection fraction (LVEF 35%) (Table 1).The patient presented a STS PROM of 2.937% and EuroSCORE II of 5.32%. The heart team decision was to treat first the AAA with an endovascular repair followed by transcatheter aortic valve replacement. The AAA was excluded percutaneously with an endoprosthesis conical, 26 × 21 mm, long 10 cm implantation (GORE TGM 26-21-10) previous aneurysmatic sac embolization by insertion of two long coils (15 mm × 250 mm) (Fig. 4, Case 5, panel C). The procedural fluoroscopic time was 19.04 min. The total amount of contrast administrated was 125 mL. One year later, the TAVR procedure was performed with a 26 mm size balloon-expandable MYVAL aortic valve bioprostheses implantation (Meril Life Sciences, Vapi, India) with 20 mm balloon pre-predilation, under rapid pacing (Table 4). The introducer advancement into the right iliac-femoral arteries (14F expandable Python introducer, Meril, Vapi, India) was uneventful. Hemostasis of the main femoral vascular access was obtained with n = 2 Proglide device (‘preclosing method’). The vascular protection with a 0.018″ guidewire was achieved through a femoral crossover contralateral access. The final angiography showed a correct valve implantation with no significant residual transvalvular gradient nor paravalvular leak (Fig. 4, Case 5, panel D). The procedural fluoroscopic time was 20.41 min. The total amount of contrast administrated was 150 mL. The pre-discharge TTE documented a transvalvular mean gradient of 10 mmHg with a slight increase in LV function (LVEF 39%) (Table 5).

## Discussion

Symptomatic AS and AAA are critical clinical conditions, increasingly more prevalent with aging of the population. TAVR is a treatment of choice in high risk and inoperable patients with symptomatic severe AS, who are often elderly [1, 6]. The symptomatic severe aortic valve stenosis (AS) in intermediate-to-high surgical risk patients is currently treated with transcatheter aortic valve replacement (TAVR) (Class I Indication according to International Guidelines) [1, 2, 11]. A continuous increase in the number of TAVR procedures-per-year is expected due to the increasingly robust evidence that TAVR is advantageous in the population with non-increased surgical risk [12].

The incidence of AAA increases with increasing age and its rupture is associated with significantly high morbidity and mortality rates. The risk of rupture increases with increasing diameter. The annual risk of rupture in patients with AAA between 5.0 and 6.9 cm is 3.0–20.0%; in patients with a AAA greater than 7.0 cm carry a high risk of rupture at 20.0% per year [13]. The standard of care requires repair of AAA prophylactically to prevent rupture in patients who are deemed appropriate candidates [14]. EVAR has shown to improve short-term morbidity and mortality, as compared to open repair, without any difference in long-term survival [15–18]. EVAR has the advantage to avoid the need for exploratory laparotomy with associated fluid shifts. It also avoids the need for aortic cross clamping with associated hemodynamic changes. Operative blood loss is lower with EVAR. EVAR can be performed with either femoral cut-downs or percutaneous femoral access under local anesthetic and is physiologically less stressful to the body. EVAR has become the mainstay of treatment for the majority of AAA, in patients with favorable neck anatomy, and has enabled patients who are unfit for surgery to undergo repair with acceptable results [19].

Both of these disease entities can be life threatening. It has been shown that up to 6% of patients with AS may have association with AAA [20, 21]. Before the advent of minimally invasive procedures, the only treatment options for the treatment of AS and AAA were open aortic valve replacement (AVR) and open abdominal aortic aneurysm repair respectively. Previously open simultaneous aneurysm repair and cardiac surgery has been advocated in highly selected patients [22]. Conventional treatment, with surgical replacement of the aortic valve and simultaneous approach of the AAA, especially in elderly patients, carries an elevated risk. In the same way, treatment of AAA in the presence of critical AS involves an unacceptably high risk. The emergence of endovascular techniques has changed the face of modern surgery with an increasing numbers of patients being treated with TAVR for severe aortic stenosis and EVAR for AAA. Nevertheless, the presence of both conditions, especially in patients with multiple comorbidities, represents a “therapeutic dilemma”.

The prior landmark clinical trials excluded patients with aortic aneurysms of more than 5.0 cm [1, 6] and the incidence of aortic aneurysms and their clinical significance was not addressed [23]. In a relatively recent study of 232 patients who underwent TAVR the incidence of concomitant aortic aneurysms was 9.5% (AAA was 6.0% and thoracic aortic aneurysm—TAA—was 4.7%) and the presence of aortic aneurysms either in the thoracic or abdominal aorta did not carry any additional risk to TAVR procedures in regards to vascular complications or short- and long-term outcomes but the average diameter of AAA was relatively small, which may have masked the true differences in the procedural and clinical outcomes between those with and without aortic aneurysms (only one patient with AAA greater than 5 cm) [20]. Certainly, the surgical or endovascular correction of AAA in the presence of severe AS could be complicated by serious hemodynamic instability, arrhythmias and secondary myocardial ischaemia and untreated severe AS increases the risk of perioperative myocardial infarction during AAA repair [24, 25]. It should also be considered that the early hemodynamic consequence of the successful TAVR is increased systolic pressure, and therefore, increased aortic vessel tension and the risk of aneurysm dilation and sudden rupture [26, 27] (Additional file 14: Movie S14).

### The rationale for simultaneous vs staged approaches

So far, individual case reports or case series of a few patients have been published for a total of less than 20 patients. Wilmo C. Orejola et al. described a case of an elderly patient with severe AS and an infrarenal saccular AAA. The principal comorbidities were coronary artery bypass grafting and mitral valve replacement (St. Jude Medical mechanical valve) and multiple percutaneous coronary interventions with stents, history of myocardial infarction, hypertension, hyperlipidemia, biventricular AICD implantation, chronic renal insufficiency. The patient underwent uncomplicated transfemoral TAVR and then EVAR in one setting [28]. Dimitrios Koudoumas et al. described a case of a 74-year-old diabetic male with chronic kidney disease, chronic obstructive pulmonary disease, cerebrovascular disease, status post left superficial temporal to middle cerebral artery bypass, left carotid endarterectomy and subsequent stent placement, 3-vessel coronary artery disease status post coronary artery bypass graft and non small cell right lung cancer stage IIIA status post induction chemoradiation with response and downstage to stage IIA, was referred for an expanding infrarenal abdominal aortic aneurysm measuring 5 cm (increased from 4 cm in 6 months). Echocardiogram showed severe AS with mean gradient of 43 mmHg, aortic orifice size of 0.48 cm^2^, mild aortic and mitral insufficiency, ejection fraction of 30–35% with hypokinesis of anterior apical region and mild pulmonary hypertension. The all five bypass grafts were patent. In the presence of his complicated medical and surgical history he was deemed high risk candidate for surgical aortic valve replacement with an STS PROM calculated at 6.2% and decision was made to proceed with simultaneous TAVR and EVAR to address both the AS and AAA [29]. Faisal Aziz et al. described simultaneous successful TAVR and EVAR procedure in a 94-year-old female with severe aortic stenosis and a large, infrarenal abdominal aortic aneurysm [30].

From the analysis of the data reported in the literature, the patients treated were extremely fragile either due to very old age or due to cardiovascular, cerebrovascular and oncological comorbidity profile. Schizas N et al. recently summarized the experience of simultaneous TAVR with EVAR [31]. The advantage of performing both operations at the same time is that they can be performed using the same access site and without the need for a second anesthesia for the second operation. The main disadvantages of the combined procedure are the increase in operative time, a higher dose of (combined) contrast and intravenous heparin.

Our ‘tailored’ interventional approach, discussed and approved by the local Heart Team for each patient, has planned an endovascular correction of the AAA as the first step, followed by a ‘staged’ TAVR. The AAA was treated first because was it considered strategically better in the presence of severe AS in stable clinical conditions. Moreover the potential elevation of the systolic arterial pressure after TAVR might provoke enhanced strain at the AAA wall increasing the risk of rupture [32–34]. During the EVAR procedure all the material needed to proceed to an ‘urgent’ aortic valvuloplasty or TAVR if necessary was available and ready.

The proposed treatment was effective in all treated patients, offering the resolution of both problems through totally percutaneous treatment in patients with a significant high risk for both mortality and vascular complications. TAVR treatment through EVAR has not been extensively studied and it is not free from potential severe complications related to the risk of malposition, embolization and/or displacement of the previously positioned aortic endoprosthesis. This approach is feasible and safe; in fact, in our series the mortality was 0%. From case to case it is necessary to use guidewires with high support forces, long introducers of adequate caliber and more rarely artero-arterial rail. The risk of acute kidney injury is reduced compared to simultaneous procedures because the total amount of contrast agents used overall is spread among two different procedures after an adequate time of recovery. The transfemoral approach was the most viable option. An alternative route, such as trans-subclavian artery, transapical and transaortic approaches for TAVR, was not used. Transcaval approach might be considered but it is not feasible in the presence of an AAA.

The main clinical, technical and procedural considerations of TAVR procedure in this case series were:

- Transfemoral access into the bifurcated endoprosthesis was feasible in 100% of patients
- The use of highly supportive stiffer guides (or buddy wire technique) to overcome the tortuosity of the iliac-femoral approaches should be considered;
- The introducer advancement under fluoroscopic guidance and through-and-through wire technique is strongly recommended in the presence of tortuosity;
- The strategy to protect the main vascular access must be personalized choosing between the ipsilateral femoral approach or the radial (or omeral) approach;
- The total amount of contrast medium administered to patients should be carefully monitored;
- The procedures should always be conducted with as much 'contrast zero' methods as possible.
- Define a delay time between EVAR and TAVR according to the clinical status, the age and the co-morbidities of patients even in the presence of clinical stability, is highly debated.

## Conclusions

The combined 'staged' EVAR and TAVR, in patients with severe AS and a large AAA, is feasible, safe and with acceptable risks in this consecutive series of cases performed in our CardioThoracic Center, especially in regards to the vascular complications and acute kidney injury. The sequence of procedures proposed, however, should be applied to a larger population in order to define the correct approach and to draw any conclusion.

In our experience, the EVAR procedure, always conducted in a totally percutaneous manner, has always been well tolerated by patients without inducing hemodynamic instability, need for intubation or unplanned valve procedures (aortic valvuloplasty or TAVR). TAVR procedures have had 100% procedural success by totally percutaneous transfemoral approach with both self-expandable and balloon-expandable prostheses with 0% mortality.

## Supplementary Information

**Additional file 1: Movie S1.** Basal aortography in the ‘virtual basal plane’ view (‘co-planar’ view) (2° right anterior oblique (RAO) 14° caudal projection) (Case 1).

**Additional file 2: Movie S2.** Final aortography documented a slightly deep PORTICO transcatheter valve implantation with mild leak and abolition of transvalvular pressure gradient (Case 1).

**Additional file 3: Movie S3.** Control angiography after TAVR demonstrated stable complete endovascular exclusion of abdominal aortic aneurysm (AAA) with Gore bifurcated prosthesis without any endoleaks (Case 1).

**Additional file 4: Movie S4.** Control angiography after TAVR demonstrated a correct endovascular exclusion of the iliac aneurysm with Gore Excluder IBE ("Iliac Branch Endoprosthesis") (Case 1).

**Additional file 5: Movie S5.** Effective access-site haemostasis and intact perfusion of femoral artery was confirmed by final angiography after sheath removal (Case 1).

**Additional file 6: Movie S6.** From the ipsilateral femoral artery a selective angiography was performed in a posterior-anterior projection to confirm the femoral artery puncture (Case 2).

**Additional file 7: Movie S7.** Basal aortography in the ‘virtual basal plane’ view (‘co-planar’ view) (8° right anterior oblique (RAO) 13° caudal projection) (Case 2).

**Additional file 8: Movie S8.** 14F expandable eSheath advancement under fluoroscopy guidance through the Gore prosthetic elements (Case 2).

**Additional file 9: Movie S9.** Final aortography documented a correct SAPIEN 3 valve implantation without paravalvular leak and abolition of transvalvular pressure gradient (Case 2).

**Additional file 10: Movie S10.** Advancement of balloon-expandable crimped MYVAL THV under fluoroscopy guidance through the Gore prosthetic elements (Case 4).

**Additional file 11: Movie S11.** Final aortography documented a precise 24.5 mm MYVAL implantation without paravalvular leak (Case 4).

**Additional file 12: Movie S12.** Embolization of the aneurysmatic sac by insertion of two long coils (15 mm × 250 mm) after Gore endoprosthesis implantation (GORE TGM 26–21-10) (Case 5).

**Additional file 13: Movie S13.** Final aortography documented a correct 26 mm MYVAL implantation without paravalvular leak (Case 5).

**Additional file 14: Movie S14.** Fluoroscopy showed systolic pressure distension of Gore endoprosthesis after effective aortic valvuloplasty. The increased systolic pressure is able to significantly stretch the metal frame of the aortic prosthesis (personal data of authors; unpublished movies).

## Data Availability

Data were collected in our internal clinic database. All data generated or analysed during this study is included in this article and in ‘supplementary materials’.
